# Elevated BAFF Levels in the Cerebrospinal Fluid of Patients with Neuro-Behçet's Disease: BAFF is Correlated with Progressive Dementia and Psychosis

**DOI:** 10.1111/j.1365-3083.2012.02694.x

**Published:** 2012-06

**Authors:** Y Sumita, Y Murakawa, T Sugiura, Y Wada, A Nagai, S Yamaguchi

**Affiliations:** * Department of Rheumatology, Shimane University Faculty of MedicineShimane, Japan; † Department of Laboratory Medicine, Shimane University Faculty of MedicineShimane, Japan; ‡ Department of Neurology, Shimane University Faculty of MedicineShimane, Japan

## Abstract

Neuro-Behçet's disease (NBD) is a serious complication of Behçet's disease. Generally, NBD patients with a chronic course are refractory to immunosuppressive treatment, resulting in the deterioration of personality. In this study, levels of B cell-activating factor belonging to the TNF family (BAFF) were measured in the cerebrospinal fluid (CSF) from 18 patients with NBD, 27 patients with epidemic aseptic meningitis (AM), 24 patients with multiple sclerosis (MS) and 34 healthy controls. BAFF levels in patients with NBD were significantly elevated compared with healthy controls, but showed no statistically significant elevation compared with either of the disease controls. In contrast, CSF IL-6 levels were slightly elevated in patients with NBD and significantly elevated in patients with AM and MS compared with healthy controls. Patients with NBD were subdivided into two groups according to their clinical course (eight patients with a slowly progressive course presenting with psychosis and dementia and 10 patients with an acute course including aseptic meningitis, brainstem involvement and myelopathy). BAFF levels were significantly increased in those with a slowly progressive course compared with those with an acute course. CSF BAFF levels did not correlate with serum BAFF levels, CSF cell counts or CSF IL-6 levels in patients with NBD. These data suggested that BAFF was produced within the central nervous system and may be associated with the development of NBD, particularly with a progressive course.

## Introduction

Behçet's disease (BD) is a systemic disease characterized primarily by recurrent oral and genital aphthous ulcers, uveitis and skin findings. Involvement of the central nervous system (CNS), referred to as neuro-Behçet's disease (NBD), is less frequent than the other major presentations, but is one of the most serious complications of BD. NBD can occur either as a result of inflammation within the CNS or of vascular complications. NBD shows a variety of manifestations, including aseptic meningitis, increased intracranial pressure, meningomyelitis, brainstem involvement, pyramidal signs, cerebellar signs, sensory signs, and psychosis or dementia [1–3]. The most serious symptom is slowly progressive dementia or psychosis, which is resistant to immunosuppressive treatments and can result in severe deterioration of the patients’ personality. Few immunological markers are known to be associated with the activity or pathogenesis of NBD [4, 5].

B cell-activating factor belonging to the tumour necrosis factor family (BAFF) plays a role in the maturation, proliferation and survival of B cells and in T cell costimulation [6–8]. BAFF is produced by monocytes, macrophages, dendritic cells [9], neutrophils [10] and cytokine-stimulated epithelial cells [11, 12]. Accumulating evidence suggests that BAFF plays an important role in the pathogenesis of immune diseases, including systemic lupus erythematosus (SLE), lymphoid cancer, Sjögren's syndrome and multiple sclerosis (MS) [11–15]. Recent reports showed that BAFF is also constitutively expressed at the transcriptional level in normal human brain [14]. In some diseases, such as MS or B-cell lymphoma with primary CNS involvement, BAFF protein is expressed by astrocytes in the brain parenchyma and in the perivascular area, and locally increased production of BAFF may contribute to B-cell focal survival [14].

A previous report showed that IL-6 may be associated with slowly progressive NBD [4]. Therefore, we investigated cerebrospinal fluid (CSF) BAFF and IL-6 levels in patients with NBD, to determine whether they are associated with certain neuropsychiatric manifestations.

## Patients and methods

### Patients

Eighteen patients with BD (12 male and six female; mean age ± SD, 44.2 ± 13.9 years) who presented with CNS manifestations were enrolled in the study. All the patients previously had, or were concurrently showing, at least three of the four major symptoms of BD, that is, uveitis, recurrent oral aphthous ulcers, genital ulcers and skin lesions (erythema nodosum, folliculitis or subcutaneous thrombophlebitis), and fulfilled the International Study Group criteria for the diagnosis of BD [16]. The diagnosis of NBD was made at the Department of Rheumatology, Shimane University Faculty of Medicine, with care taken to rule out other differential diagnoses, including bacterial, fungal and viral infection of the CNS, cerebrovascular disease owing to atherosclerosis, metabolic disorders and other collagen diseases. As summarized in Table 1, the time from the onset of BD to diagnosis of NBD ranged from 0 to 35 years. CSF samples were collected at the onset of NBD, and after diagnosis, all patients received immunosuppressive agents to treat the CNS manifestations. Depending on the response to therapy and their clinical course, 18 patients with NBD were retrospectively subdivided into two groups: (1) those with an acute disease course and (2) those with chronic disease course. Those with an acute disease course included 10 patients with transient neurological symptoms, such as meningitis and paralysis, who rapidly responded to immunosuppressive therapy. Those with a chronic disease course contained eight patients with neuropsychiatric symptoms, such as progressive psychosis and dementia, which persisted for at least 1 year after the start of therapy (Tables 1 and 2). Of eight patients with a chronic course, three (Pt No. 11–13) showed only a partial recovery and five (Pt No. 14–18) continued to develop neuropsychiatric symptoms despite immunosuppressive therapy (Table 2).

**Table 1:** Clinical background of the patients with NBD

	Symptom										
				
Patient no.	Age at NBD onset	Sex	Oral ulcer	Genital ulcer	Uveitis	Skin lesion	Other symptoms	Neurological symptom	NBD onset after BD diagnosis	Brain MRI	Therapy for NBD
1	16	F	(+)	(+)	(−)	(+)	Arthritis Thrombophlebitis	Meningitis	Same time	WNL	PSL 60 mg/day, mino 200 mg/day
2	49	F	(+)	(−)	(+)	(+)		Myelopathy	1 year later	WNL	mPSL pulse×2
3	40	M	(+)	(−)	(+)	(+)	Arthritis	Optic neuritis	1 year later	WNL	PSL 30 mg/day, MTX 5 mg/week
4	49	M	(+)	(+)	(+)	(+)	Colon ulcer	Meningitis	9 years later	WNL	COL 1.5 mg/day, PSL 30 mg/day
5	44	F	(+)	(+)	(+)	(+)		Optic neuritis	6 years later	WNL	PSL (maximum dose): unknown, COL 1.5 mg/day, MTX 4 mg/week
6	60	F	(+)	(+)	(−)	(+)	Arthritis	Meningitis	24 years later	WNL	PSL 20 mg/day, COL 1.0 mg/day
7	34	M	(+)	(+)	(+)	(+)		Meningitis, headache	14 years later	WNL	PSL (maximum dose): unknown, COL 1.0 mg/day
8	49	M	(+)	(−)	(+)	(+)	Arthritis	Left hemiparesis	7 years later	Unknown	mPSL pulse, IVCY, MTX 8 mg/w, COL 1.5 mg/day
9	49	M	(+)	(−)	(+)	(+)	Arthritis	Meningitis, dizziness	17 years later	Old infarction in cerebellum	mPSL pulse, MTX 4 mg/week
10	24	M	(+)	(−)	(+)	(+)	Colon ulcer	Meningitis	2 months later	High intensity in the medulla	mPSL pulse
11	49	F	(+)	(+)	(+)	(+)	Colon ulcer	Dementia	8 months later	High intensity in the white matter	PSL 60 mg/day, MTX 4 mg/week
12	31	F	(+)	(+)	(−)	(+)	Arthritis	Psychosis, rt sensory distuebance	17 years later	High intensity in the white matter, rt thalamus, pons, Lt cerebellum	mPSL pulse, COL 1.0 mg/day
13	67	M	(+)	(+)	(+)	(+)	Colon ulcer, Thrombophlebitis	Psychosis, meningitis	42 years later	WNL	PSL 30 mg/day, COL 1.0 mg/day
14	67	F	(+)	(+)	(+)	(+)	Arthritis	Confusion, dementia	33 years later	WNL	PSL 45 mg/day
15	30	M	(+)	(+)	(+)	(+)	Arthritis	Pcychosis	8 years later	WNL	PSL 60 mg/day, COL 1.0 mg/day
16	56	M	(+)	(+)	(+)	(+)	Colon ulcer, Thrombophlebitis	Psychosis, meningitis	35 years later	WNL	PSL (maximum dose); unknown, COL 1.0 mg/day
17	35	M	(+)	(−)	(+)	(+)		Dementia, psychosis	1 year later	WNL	PSL 60 mg/day, MTX 4 mg/week, COL 1.0 mg/day
18	30	M	(+)	(+)	(+)	(+)		Dementia, psychosis	6 years later	High intensity in the white matter	PSL 50 mg/day, MTX 4 mg/week, COL 1.5 mg/day

NBD, Neuro-Behçet's disease; PSL, prednisolone; COL, colchicine; MTX, methotrexate; mino, minocycline; IVCY, intravenous cyclophosphamide pulse therapy; rt, right; Lt, left.

**Table 2:** Profiles and clinical data for the patients with NBD

					CSF findings at diagnosis			CSF findings after therapy		
						
NBD course	Patient no.	Psycosis	Dementia	Neurological symptoms after therapy	BAFF (ng/ml)	Cell counts (/mm^3^)	IL-6 (pg/ml)	BAFF (ng/ml)	Cell counts (/mm^3^)	IL-6 (pg/ml)
Acute	1	−	−	Improved	<0.31	160	83.6	<0.31	4	1.1
Acute	2	−	−	Improved	<0.31	43	0.7	<0.31	5	0.9
Acute	3	−	−	Improved	<0.31	1	1.2	<0.31	1	0.8
Acute	4	−	−	Improved	<0.31	53	14.6	<0.31	8	2.1
Acute	5	−	−	Improved	<0.31	3	0.8	ND	ND	ND
Acute	6	−	−	Improved	6.1	57	20.3	ND	ND	ND
Acute	7	−	−	Improved	6.3	8	0.9	ND	ND	ND
Acute	8	−	−	Improved	7.5	35	0.6	8.9	4	1.1
Acute	9	−	−	Improved	12.4	14	37.7	10.6	10	3.2
Acute	10	−	−	Improved	5.1	12	1.1	ND	ND	ND
Chronic	11	−	+	Partially improved	5.4	8	0.3	<0.31	3	ND
Chronic	12	+	−	Partially improved	5.4	37	1	4.8	2	0.4
Chronic	13	+	−	Partially improved	10.7	6	0.9	6.2	0	1.5
Chronic	14	+	+	Progressive	9.3	84	244.3	3.2	3	ND
Chronic	15	+	−	Died (suicide)	11.2	9	0.6	8.9	5	0.9
Chronic	16	+	−	Progressive	10.1	3	2.1	5.6	6	1
Chronic	17	+	+	Progressive	17.9	7	1.7	11.4	12	1.8
Chronic	18	+	+	Progressive	11.6	2	1	9.2	1	1

CSF, cerebrospinal fluid; ND, no data; NBD, Neuro-Behçet's disease.

### CSF and serum samples and ethics

All CSF samples were collected from patients with NBD (*n* = 18) with the aim of diagnosis or to estimate therapeutic effects, and informed consent was obtained from all patients. In 14 of 18 patients with NBD, repeated CSF sampling was performed after immune suppressive therapy, and CSF parameters were compared with those obtained before treatment.

Cerebrospinal fluid samples were obtained from the following patients as disease controls: 27 patients (16 males and 11 females; mean age ± SD, 30.1 ± 8.5 years) with epidemic aseptic meningitis (AM; possibly because of viral infection) and 24 patients (13 males and 11 females; mean age ± SD, 39.8 ± 12.9 years) with MS. All AM patients received conservative therapy, but no immunosuppressive therapy. The 24 patients with MS included 17 patients with remitting type, three patients with primary progressive type and four patients with secondary progressive type. After CSF sampling, all patients with MS received immunosuppressive therapy (oral glucocorticoid therapy or methyl-prednisolone pulse therapy).

Cerebrospinal fluid samples from 34 normal controls (17 men and 17 women; mean age ± SD, 55.9 ± 21.7 years) were obtained by conventional lumbar puncture during plastic surgery after informed consent.

Cell counts (normal; ≤3/mm^3^) in the CSF were measured using routine laboratory procedures. The CSF samples were centrifuged to remove the cell fraction and kept frozen at −70 °C until use. Concentrations of BAFF and IL-6 were measured by ELISA. The study protocol was approved by the Ethics Committee of Shimane University Faculty of Medicine.

Serum samples were obtained from nine of the 18 patients with NBD (acute type; chronic type = 4:5) before the initiation of immunosuppressive therapy, and serum BAFF and IL-6 concentrations were measured by ELISA. Sera from 24 healthy individuals served as controls, although these individuals were not the same as those used for CSF controls.

### Enzyme-linked immunosorbent assay (ELISA)

The levels of BAFF and IL-6 in the CSF and sera were measured in triplicate using commercial ELISA kits (Bender MedSystems, Vienna, Austria, for BAFF; high-sensitivity IL-6 ELISA, R&D Systems, Minneapolis, MN, USA, for IL-6). The detection ranges were 0.31–20 ng/ml for BAFF and 0.156–10 pg/ml for IL-6. The intra-assay variability of the ELISA was <3%, and the inter-assay variability was <5%.

### Statistical analyses

The Kruskal–Wallis test was used to compare BAFF and IL-6 concentrations in CSF from the four groups. The criteria for the *post hoc* test were adjusted using the Bonferroni correction. Pearson's correlation coefficient was used to ascertain whether correlations existed between any two parameters. Wilcoxon's test was applied for the comparison of the paired samples. A *P-*value of <0.05 was considered statistically significant.

## Results

### BAFF and IL-6 levels in the CSF and sera

**Figure 1:**
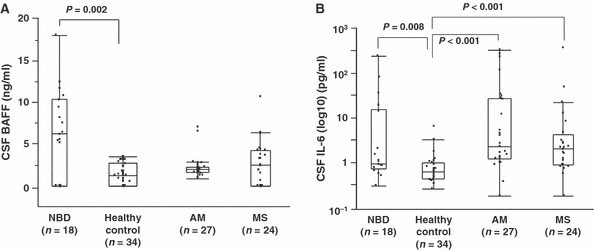
(A) BAFF levels in cerebrospinal fluid (CSF) from patients with neuro-Behçet's disease (NBD) at diagnosis, aceptic meningitis (AM), multiple sclerosis (MS) and healthy controls were measured by ELISA. CSF BAFF levels were significantly higher in patients with NBD (mean ± SD, 6.4 ± 5.1 ng/ml; median, 6.1 ng/ml) compared with healthy controls (mean ± SD, 1.4 ± 1.2 ng/ml; median, 1.2 ng/ml). The Kruskal–Wallis test showed a significant main group effect (*P* < 0.01), and the *post hoc* test showed that BAFF levels in NBD were higher than those in the healthy control group (*P* = 0.002). There was no significant difference between the patients with NBD and the disease controls including AM (mean ± SD, 2.2 ± 1.4 ng/ml; median, 1.9 ng/ml) and MS (mean ± SD, 2.7 ± 2.6 ng/ml; median, 2.4 ng/ml). Results are shown as box plots. Each box represents the 25th and 75th percentiles. Lines outside the boxes represent the 10th and the 90th percentiles. Lines inside the boxes represent the median. (B) CSF IL-6 levels in patients with NBD (mean ± SD, 23.0 ± 59.0 pg/ml; median, 1.1 pg/ml), healthy control subjects (mean ± SD, 1.1 ± 1.3 pg/ml; median, 0.7 pg/ml), patients with AM (mean ± SD, 41.3 ± 88.8 pg/ml; median, 2.4 pg/ml) and patients with MS (mean ± SD, 20.5 ± 73.6 pg/ml; median, 2.2 pg/ml). The Kruskal–Wallis test showed a significant difference as indicated. The *post hoc* test indicated CSF IL-6 levels in NBD were elevated compared with those in healthy controls with a borderline significance (*P* = 0.008), and CSF IL-6 levels in AM and MS were significantly higher than those in healthy controls (NBD; *P* = 0.008, AM and MS; *P* < 0.001).

Figure 1A shows a comparison of BAFF levels in the CSF between the four groups (18 patients with NBD, 27 patients with AM, 24 patients with MS and 34 normal controls). The Kruskal–Wallis test showed a significant effect between the groups (*P* < 0.01), and the *post hoc* test showed that the BAFF levels in the CSF of patients with NBD (mean ± SD, 6.4 ± 5.1 ng/ml; median, 6.2 ng/ml) were significantly higher than the normal control group (mean ± SD, 1.4 ± 1.2 ng/ml; median, 1.2 ng/ml), (*P* = 0.002). There were no statistically significant differences in the CSF BAFF levels between patients with NBD and the other disease controls, including AM (mean ± SD, 2.2 ± 1.4 ng/ml; median, 1.9 ng/ml) and MS (mean ± SD, 2.7 ± 2.6 ng/ml; median, 2.4 ng/ml).

Comparison of the CSF IL-6 concentrations in the four groups (Fig. 1B) showed the mean CSF IL-6 level in patients with NBD was 23.0 ± 59.0 pg/ml (median, 1.1 pg/ml), which was higher than that of healthy controls with a borderline significance (*P* = 0.008). Besides NBD, the *post hoc* test indicated significant differences between patients with AM (mean ± SD, 41.3 ± 88.9 pg/ml; median, 2.4 pg/ml) and healthy controls (mean ± SD, 1.1 ± 1.3 pg/ml; median, 0.7 pg/ml), (*P* < 0.001) and between patients with MS (mean ± SD, 20.5 ± 73.6 pg/ml; median, 2.2 pg/ml) and controls (*P* < 0.001).

Serum samples were obtained from nine of 18 patients with NBD (four with acute course and five with chronic course) before the initiation of therapy. There was no difference between serum BAFF levels in patients with NBD at diagnosis (mean ± SD, 7.9 ± 7.6 ng/ml; median, 5.5 ng/ml) and control subjects (mean ± SD, 10.5 ± 4.8 ng/ml; median, 10.2 ng/ml). There was also no difference between serum IL-6 levels in patients with NBD (mean ± SD, 41.8 ± 59.5 pg/ml; median, 15.2 pg/ml) and control subjects (mean ± SD, 36.7 ± 63.9 pg/ml; median, 15.8 pg/ml).

### Association of CSF BAFF levels with serum BAFF level and CSF cell parameters in NBD

**Figure 2:**
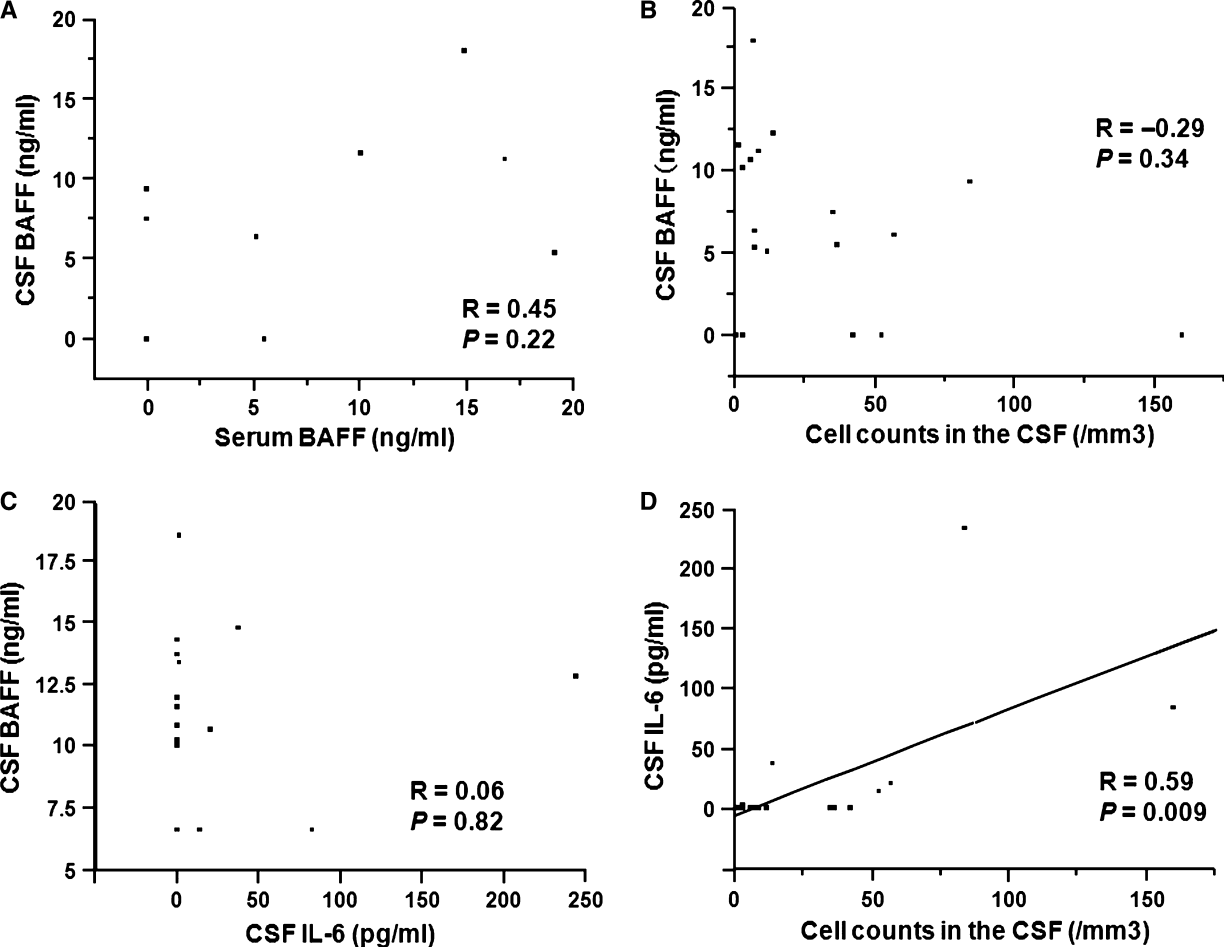
(A) No significant correlation was detected between cerebrospinal fluid (CSF) BAFF levels and serum BAFF levels in patients with Neuro-Behçet's disease (NBD) whose sera were available (*n* = 9, *r* = 0.45, *P* = 0.22). (B) CSF BAFF levels in NBD at diagnosis did not correlate with CSF cell counts (*n* = 18, *r* = −0.29, *P* = 0.34). (C) Correlation analysis between CSF BAFF and IL-6 levels in 18 patients with NBD. There was no association between the two parameters (*n* = 18, *r* = 0.06, *P* = 0.82). (D) Correlation between CSF IL-6 levels and CSF cell counts in 18 patients with NBD. There was a significant association between CSF IL-6 levels and CSF cell counts (*r* = 0.59, *P* = 0.009).

As shown in Fig. 2A, no significant correlation was detected between CSF and serum BAFF levels in patients with NBD whose sera were available (*n* = 9, *r* = 0.45, *P* = 0.22). Next, we examined whether CSF BAFF levels correlated with the intensity of inflammation estimated by routine CSF cell counts in patients with NBD. CSF BAFF levels did not correlate with CSF cell counts (*r* = −0.29, *P* = 0.34, Fig. 2B). Furthermore, no association was found between CSF BAFF and CSF IL-6 levels in NBD (*r* = 0.06, *P* = 0.82, Fig. 2C). In contrast, IL-6 levels significantly correlated with CSF cell counts (*r* = 0.59, *P* = 0.009, Fig. 2D).

### BAFF levels in patient with NBD grouped according to clinical course

**Figure 3:**
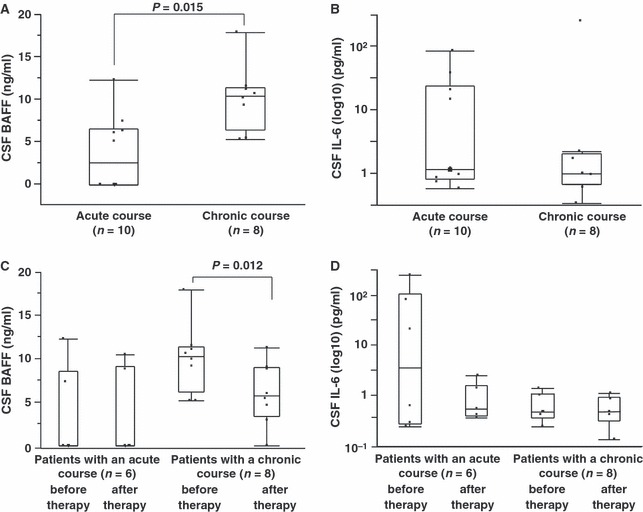
(A) Comparison of BAFF levels in the cerebrospinal fluid (CSF) and clinical subsets of Neuro-Behçet's disease (NBD). CSF BAFF levels were significantly higher in patients with a chronic course (*n* = 8) compared with those with an acute course (*n* = 10), (mean ± SD, 10.2 ± 4.0 ng/ml; median, 10.4 ng/ml versus mean ± SD, 3.9 ± 4.2 ng/ml; median, 2.7 ng/ml, *P* = 0.015). (B) CSF IL-6 levels were not significantly different between patients with a chronic course and those with an acute course (mean ± SD, 31.5 ± 86.0 pg/ml; median, 1.0 pg/ml versus mean ± SD, 16.2 ± 26.7 pg/ml; median, 1.1, *P* = 0.65). (C) Of 18 patients with NBD, 14 (eight patients with a chronic course and six patients with an acute course), paired CSF samples were obtained before and after therapy. In NBD patients with a chronic course (*n* = 8), BAFF levels significantly decreased from mean ± SD of 10.2 ± 4.0 ng/ml and median of 10.4 ng/ml to 6.2 ± 3.6 ng/ml and 5.9 ng/ml after therapy, respectively (*P* = 0.012, Wilcoxon's test). Of six of NBD patients with an acute course whose sera were available, BAFF levels at diagnosis were not different from those after therapy (mean ± SD, 3.5 ± 5.2 ng/ml; median, 0.3 ng/ml versus mean ± SD, 3.5 ± 4.9 ng/ml; median, 0.3 ng/ml). (D) Changes in CSF IL-6 levels before and after therapy. There was no difference in CSF IL-6 levels between time of diagnosis and after therapy, both in patients with an acute course (mean ± SD, 23.0 ± 32.9 pg/ml; median, 7.9 pg/ml at diagnosis and mean ± SD, 1.5 ± 0.9 pg/ml; median, 1.1 pg/ml after therapy) and in patients with a chronic course (from mean ± SD, 1.2 ± 0.6 pg/ml; median, 1.0 pg/ml at diagnosis to 1.1 ± 0.5 and 1.0 pg/ml after therapy).

Patients with NBD were subdivided into two groups depending on their response to therapy: 10 patients with an acute course who responded to immunosuppressive therapy and eight patients with a chronic course who presented psychosis and dementia. As shown in Fig. 3A, CSF BAFF levels at diagnosis were significantly higher in patients with a chronic course (*n* = 8, mean ± SD, 10.2 ± 4.0 ng/ml; median, 10.4 ng/ml) compared with those with an acute course (*n* = 10; mean ± SD, 3.9 ± 4.2 ng/ml; median, 2.7 ng/ml, *P* = 0.015).

All 18 patients with NBD received immunosuppressive therapies as summarized in Table 1, and CSF samples were obtained by repeated lumbar puncture after immunosuppressive treatments in six of 10 NBD patients with an acute course and eight NBD patients with a chronic course. Fourteen paired CSF samples were obtained before and after therapy. In NBD patients with a chronic course (*n* = 8), BAFF levels significantly decreased from 10.2 ± 4.0 ng/ml (median, 10.4 ng/ml) to 6.2 ± 3.6 ng/ml (median, 5.9 ng/ml) after therapy (*P* = 0.012, Wilcoxon's test, Fig. 3C). In NBD patients with an acute course in whom paired samples were obtained (*n* = 6), BAFF levels at diagnosis were not different from those after treatment (mean ± SD, 3.5 ± 5.2 ng/ml; median, 0.31 ng/ml versus mean ± SD, 3.5 ± 4.9 ng/ml; median, 0.31 ng/ml), (Fig. 3C). As shown in Fig. 3D, there was no difference between CSF IL-6 levels at diagnosis and after therapy, either in patients with an acute course (mean ± SD, 23.0 ± 32.9 pg/ml; median, 7.9 pg/ml at diagnosis and mean ± SD, 1.5 ± 0.9 pg/ml; median, 1.1 pg/ml after therapy) or with a chronic course (mean ± SD, 1.2 ± 0.6 pg/ml; median, 1.0 pg/ml at diagnosis and mean ± SD, 1.1 ± 0.5 pg/ml; median, 1.0 pg/ml after therapy).

## Discussion

The results from this study showed that BAFF levels in CSF were elevated in patients with NBD, and were independent of serum BAFF levels. It should be noted that NBD patients with progressive CNS manifestations such as psychosis and dementia showed significantly higher CSF BAFF levels compared with those with acute and transient CNS manifestations.

Although the pathogenesis of NBD remains unclear, elevated CSF levels of inflammatory cytokines such as IL-6, IL-8 and IL-15 have been reported [4, 5, 17]. Interestingly, the elevation of pro-inflammatory cytokines is associated with acute CNS inflammation, such as meningitis [5]. In the present study, elevated BAFF levels were associated with NBD that presented with slowly progressive CNS manifestations. Analysis of CSF IL-6 levels demonstrated significantly higher levels in the patients with NBD, AM and MS compared with healthy controls (Fig. 1B). CSF IL-6 levels correlated with CSF cell counts, but no correlation was found between CSF BAFF levels and CSF cell counts. In addition, there was no association between BAFF and IL-6 levels. Therefore, IL-6 levels in NBD might reflect acute inflammation within the CNS caused by the presence of inflammatory cells, although the elevated levels may not be specific to the disease. In contrast, increased BAFF may indicate a distinct disease state.

The key questions relate to the source of BAFF within the CNS and the contribution of BAFF to the pathogenesis of NBD, particularly in patients with progressive disease. Increased CSF BAFF levels did not correlate with serum BAFF levels or CSF cell counts, suggesting BAFF may be produced within the CNS of patients with NBD. With respect to CNS autoimmunity, a previous study in patient with MS showed that the main source of BAFF is brain glial cells, particularly astrocytes. Furthermore, cytokine-stimulated astrocytes produced higher levels of BAFF than activated monocytes or macrophages [14]. Therefore, similar to MS, astrocytes in the CNS lesions of patients with NBD may also secrete BAFF.

The contribution of BAFF to the pathogenesis of NBD is still unknown. In MS, the contribution of B cell-mediated immune responses is suggested because of the presence of oligoclonal bands and B cells and plasma cells in MS plaques [18–20]. However, the involvement of B cells in the immunopathogenesis of NBD is unclear, although oligoclonal bands have been detected in the CSF from some patients with NBD [21, 22]. Additionally, oligoclonal B-cell expansion in the synovium of a patient with BD has been observed [23]. These data suggest that B cells may play a role in the pathogenesis of NBD. Another possibility is that local BAFF production within NBD lesions may promote T cell activation, as BAFF is a costimulatory molecule for T cells [6, 7, 24]. Immunohistochemical analysis examining BAFF- and BAFF-receptor-expressing cells within NBD lesions would be helpful in further elucidating the role of BAFF.

Recent reports from experimental models highlight the possibility that inflammatory processes and cytokines in the brain contribute to the pathogenesis of mood disorders [25]. BAFF transgenic (Tg) mice show chronic inflammation in the CNS, impaired neurogenesis and an increased anxiety phenotype [26], similar to common clinical symptoms in NBD patients with a chronic course.

Immunosuppressive therapy influenced CSF BAFF levels in the present patients with NBD. All eight NBD patients with a chronic course underwent repeated CSF examination, and CSF BAFF levels significantly decreased after therapy as shown in Fig. 3C (from mean ± SD, 10.2 ± 4.0 ng/ml to mean ± SD, 6.2 ± 3.6 ng/ml; *P* = 0.012, Wilcoxon's test). This observation indicates immunosuppressive therapy would partially suppress BAFF production. However, it should be noted that NBD patients with a chronic course showed high CSF BAFF levels even after immunosuppressive treatment when compared with those in healthy controls (mean ± SD, 6.2 ± 3.6 ng/ml versus mean ± SD, 1.4 ± 1.2 ng/ml, *P* < 0.001). Sustained elevation of CSF BAFF levels reflect persistent CNS inflammation or damage and might play an important role in the pathogenesis of progressive neuropsychiatric manifestations in NBD. This might be consistent with observations that NBD patients with a chronic course generally show poor responses to any form of immunosuppressive therapy. Thus, CSF levels of BAFF may be a good prognostic marker for NBD, especially in patients with a chronic disease course.

## References

[1] Abraham S, Rajeev E, Girija AS (2000). Neuro-Behcet's disease. J Assoc Physicians India.

[2] Benamour S, Naji T, Alaoui FZ, El-Kabli H, El-Aidouni S (2006). [Neurological involvement in Behcet's disease. 154 cases from a cohort of 925 patients and review of the literature]. Rev Neurol (Paris).

[3] Sakane T, Takeno M, Suzuki N, Inaba G (1999). Behcet's disease. N Engl J Med.

[4] Hirohata S, Isshi K, Oguchi H (1997). Cerebrospinal fluid interleukin-6 in progressive Neuro-Behcet's syndrome. Clin Immunol Immunopathol.

[5] Hamzaoui K, Hamzaoui A, Ghorbel I, Khanfir M, Houman H (2006). Levels of IL-15 in serum and cerebrospinal fluid of patients with Behcet's disease. Scand J Immunol.

[6] Moore PA, Belvedere O, Orr A (1999). BLyS: member of the tumor necrosis factor family and B lymphocyte stimulator. Science.

[7] Schneider P, Mackay F, Steiner V (1999). BAFF, a novel ligand of the tumor necrosis factor family, stimulates B cell growth. J Exp Med.

[8] He B, Chadburn A, Jou E, Schattner EJ, Knowles DM, Cerutti A (2004). Lymphoma B cells evade apoptosis through the TNF family members BAFF/BLyS and APRIL. J Immunol.

[9] Nardelli B, Belvedere O, Roschke V (2001). Synthesis and release of B-lymphocyte stimulator from myeloid cells. Blood.

[10] Scapini P, Nardelli B, Nadali G (2003). G-CSF-stimulated neutrophils are a prominent source of functional BLyS. J Exp Med.

[11] Ittah M, Miceli-Richard C, Eric Gottenberg J (2006). B cell-activating factor of the tumor necrosis factor family (BAFF) is expressed under stimulation by interferon in salivary gland epithelial cells in primary Sjogren's syndrome. Arthritis Res Ther.

[12] Szodoray P, Alex P, Jonsson MV (2005). Distinct profiles of Sjogren's syndrome patients with ectopic salivary gland germinal centers revealed by serum cytokines and BAFF. Clin Immunol.

[13] Mackay F, Tangye SG (2004). The role of the BAFF/APRIL system in B cell homeostasis and lymphoid cancers. Curr Opin Pharmacol.

[14] Krumbholz M, Theil D, Derfuss T (2005). BAFF is produced by astrocytes and up-regulated in multiple sclerosis lesions and primary central nervous system lymphoma. J Exp Med.

[15] Khare SD, Sarosi I, Xia XZ (2000). Severe B cell hyperplasia and autoimmune disease in TALL-1 transgenic mice. Proc Natl Acad Sci U S A.

[16] (1990). Criteria for diagnosis of Behcet's disease. International Study Group for Behcet's Disease. Lancet.

[17] Itoh R, Takenaka T, Okitsu-Negishi S, Matsushima K, Mizoguchi M (1994). Interleukin 8 in Behcet's disease. J Dermatol.

[18] Archelos JJ, Storch MK, Hartung HP (2000). The role of B cells and autoantibodies in multiple sclerosis. Ann Neurol.

[19] Lucchinetti C, Bruck W, Parisi J, Scheithauer B, Rodriguez M, Lassmann H (2000). Heterogeneity of multiple sclerosis lesions: implications for the pathogenesis of demyelination. Ann Neurol.

[20] McLean BN, Miller D, Thompson EJ (1995). Oligoclonal banding of IgG in CSF, blood-brain barrier function, and MRI findings in patients with sarcoidosis, systemic lupus erythematosus, and Behcet's disease involving the nervous system. J Neurol Neurosurg Psychiatry.

[21] Akman-Demir G, Serdaroglu P, Tasci B (1999). Clinical patterns of neurological involvement in Behcet's disease: evaluation of 200 patients. The Neuro-Behcet Study Group. Brain.

[22] Kidd D, Steuer A, Denman AM, Rudge P (1999). Neurological complications in Behcet's syndrome. Brain.

[23] Suh CH, Park YB, Song J, Lee CH, Lee SK (2001). Oligoclonal B lymphocyte expansion in the synovium of a patient with Behcet's disease. Arthritis Rheum.

[24] Mukhopadhyay A, Ni J, Zhai Y, Yu GL, Aggarwal BB (1999). Identification and characterization of a novel cytokine, THANK, a TNF homologue that activates apoptosis, nuclear factor-kappaB, and c-Jun NH2-terminal kinase. J Biol Chem.

[25] Reichenberg A, Yirmiya R, Schuld A (2001). Cytokine-associated emotional and cognitive disturbances in humans. Arch Gen Psychiatry.

[26] Crupi R, Cambiaghi M, Spatz L (2010). Reduced adult neurogenesis and altered emotional behaviors in autoimmune-prone B-cell activating factor transgenic mice. Biol Psychiatry.

